# Gender differences in predictors of self-rated health in Armenia: a population-based study of an economy in transition

**DOI:** 10.1186/1475-9276-11-67

**Published:** 2012-11-14

**Authors:** Anahit Demirchyan, Varduhi Petrosyan, Michael E Thompson

**Affiliations:** 1 College of Health Sciences, American University of Armenia, 40 Marshal Baghramian Avenue, Yerevan 0019, Armenia; 2 Department of Public Health Sciences, University of North Carolina at Charlotte, 9201 University City Blvd, Charlotte, NC 28223-0001, USA

**Keywords:** Armenia, Self-rated health, Depression, Social support, Poverty, Psychosocial pathways

## Abstract

**Introduction:**

Self-rated health is a widely used health outcome measure that strongly correlates with physical and mental health status and predicts mortality. This study identified the set of predictors of fair/poor self-rated health in adult female and male populations of Armenia during a period of long-lasting socio-economic transition to a market economy.

**Methods:**

Differences in self-rated health were analyzed along three dimensions: socioeconomic, behavioral/attitudinal, and psychosocial. The study utilized data from a 2006 nationwide household health survey that used a multi-stage probability proportional to size cluster sampling with a combination of interviewer-administered and self-administered surveys. Both female and male representatives of a household aged 18 and over completed the self-administered survey. Multivariate odds ratios (OR) for fair/poor self-rated health were calculated for different sets of variables and logistic regression models fitted separately for women and men to identify the determinants of fair/poor self-rated health.

**Results:**

Overall, 2310 women and 462 men participated in the survey. The rate of fair/poor self-rated health was 61.8% among women and 59.7% among men. For women, the set of independent predictors of fair/poor self-rated health included age, unemployment, poverty, low affordability of healthcare, depression, and weak social support. For men, the set included age, lower education, depression, weak social support, and drinking alcohol less than once a week. For both genders, depression and weak social support demonstrated the strongest independent association with fair/poor self-rated health.

**Conclusions:**

The prevalence of fair/poor self-rated health was similar among men and women in this study, but the sets of independent predictors of perceived health differed somewhat, possibly, reflecting lifestyle differences between men and women in Armenia. Nevertheless, psychosocial variables were the strongest predictors of fair/poor self-rated health for both genders, indicating the importance of improving the country’s psychosocial environment through social reforms and poverty reduction.

## Introduction

Self-rated health is widely used in cross-sectional studies as a single-item health outcome measure that strongly correlates with objective physical and mental health status [1-5]. The predictive ability of self-rated health for mortality risk is well documented [1,6-10]. Although findings conflict as to whether the power of self-rated health in predicting mortality varies by socioeconomic group [11,12], the basic message remains unchanged: self-rated health is an independent predictor of subsequent morbidity and mortality prognosis [13,14]. It captures more than the simple absence of ill-health, covering the entire illness-wellness continuum and acting as a measure of “health optimism,” which includes fitness, healthy behavior, greater social support and less depression [3,15]. This measure also has been recommended as a disease risk screening tool [16]. Considering the easily-obtainable nature of this valid and reliable [17] health outcome measure, the World Health Organization (WHO) recommended including self-rated health as a standard component of health surveys [18].

The determinants of self-rated health have been widely investigated to explain social differences in health [19-26]. Absolute and relative poverty and their psychosocial consequences, such as low life control, depression, lack of social support and adverse health behaviors have been found to largely influence health [22,27]. Many studies investigating the reasons for declining health indicators in post-communistic countries of Central and Eastern Europe used fair/poor self-rated health as an outcome measure and found that perceived health is related to education, perceived life control and social support as well as absolute and relative poverty expressed by widening income inequalities in these countries [2,19,20,28-32].

During the years of difficult transition from the soviet system to market economy, considerable decline in health indicators was observed in Armenia as in many transition economies [33]. This small Transcaucasian country experienced a number of cataclysms that adversely effected its economy and its populations’ health status [34,35]. Since the mid 1980s, the crude mortality rate in Armenia increased by over 50% (from 5.7 per 1000 in 1986 to 8.6 per 1000 in 2010) [33,36]. Over half of the population were impoverished until the early 2000s, after which the proportion of poor started to decrease slowly (37.7% in 2009) [33,37]. However, the Gini coefficient (an index of income concentration) still exceeds the threshold value of 0.3 (0.362 in 2010), indicating a strong influence of income inequality on health outcomes [37,38].

Only one study was conducted in Armenia on determinants of self-rated health [35]. However, the study population was limited to only women living in one of Armenia’s 11 provinces (*marzes)*. The current study aims to identify the associations between fair/poor self-rated health and social structure, behavioral/attitudinal and psychosocial factors among women and men using a representative population sample from a nationwide household health survey [39]. The study aims to reveal whether the set of predictors of perceived health vary by gender, and to understand whether interventions should be aimed primarily at changes in material conditions, behavioral factors, or the psychosocial environment in order to improve the health of population in Armenia.

## Methods

### The survey

The data for this study were obtained from a country-wide household health survey conducted in 2006 in the scope of a 5-year Primary Healthcare Reform Project sponsored by the United States Agency for International Development (USAID). This cross-sectional survey utilized a probability proportional to size multi-stage cluster sampling design stratified by *marzes* to generate a sample of 2310 households throughout the country [39]. The strategies for sampling and respondent selection for this survey repeated those applied during earlier household health surveys in Armenia described elsewhere [35]. The survey consisted of interviewer-administered and self-administered questionnaires. The item on self-rated health was part of the self-administered questionnaire that was completed by a female respondent from a selected household and, whenever available at the time of interview, concurrently by a male respondent from the same household. The self-administered questionnaire also included items on respondent’s quality of life, chronic health problems, social support, depression, health-related behaviors and attitudes, living standards, and socio-demographic characteristics. The Institutional Review Board of the American University of Armenia reviewed and approved the survey protocol.

### Variables

Most variables were dichotomized to enhance the interpretability of the logistic regression coefficients. For ordinal variables, dummy variables were created with the “best” category used as the referent. Only age was treated as a continuous variable after checking its linearity on the logistic scale [40].

The outcome variable, *self-rated health,* was assessed with the question “How would you describe your health in the last 30 days?”, with the response options “excellent”, “very good”, “good”, “fair” and “poor” and then dichotomized as fair/poor versus good.

The variable of *physical health* was constructed based on a cumulative score generated from the responses to the following items: the number of self-reported chronic diseases, the extent of being limited in daily activities because of health and the extent of bodily pain experienced. This variable was divided into three categories: *severe, moderate and minimal/no health problems* to determine the dose–response association with the outcome variable.

The dimension of social structure included *age, urban/rural residence, education, ethnicity, employment, level of poverty and affordability of healthcare*. All these variables, except *level of poverty*, were based on single items. *Level of poverty* was constructed on the basis of a cumulative score generated from the responses to the following items: number of employed household members, rating of family’s general standard of living, number of possessed convenience/luxury items and household’s last month expenditures. This score was divided into three categories indicating *severe, moderate and no poverty*.

The behavioral/attitudinal dimension included *ever smoking, current smoking, moderate drinking* (consuming alcohol once a week or more), *binge drinking* (all based on single items) and *positive attitude toward healthy lifestyle* (based on a score generated from the expressed agreement to six statements and then dichotomized).

The psychosocial dimension included *depression* and *weak social support*. *Depression* was evaluated through the modified CES-D scale [41] with the use of 16 negatively worded items and a threshold level of 12/13 [42]. “*Weak social support*” was a multi-item variable based on a cumulative satisfaction score from relationships with and support from family, friends and community and then dichotomized.

### Statistical analyses

The sets of determinants of self-rated health could vary among women and men [43], thus, this analyses was stratified by gender. The variables significantly associated with fair/poor self-rated health were identified through bivariate logistic regression analysis. Then, to affirm the use of self-rated health as a valid measure of health status, we analyzed the association between self-rated health and its physical and mental correlates – *physical health* and *depression –* while first controlling only for age and then with all other significant variables.

Next, the variables found significant in the bivariate analysis were entered into a multivariate logistic regression analysis in conceptually coherent blocks in three steps. First, the “gross effect” of each variable was measured when controlling only for age (Model 1). Second, all significant social structure variables were introduced into the model (Model 2). Next, the significant variables in behavioral/attitudinal and psychosocial dimensions were added (Model 3). These successive steps allowed measuring the extent of direct and, in different combinations, mediated/controlled effect of each variable on the outcome.

The final step identified the sets of principal predictors of self-rated health in women and men, using all variables significant at the p<0.25 level in the bivariate analysis to fit the final models [40]. Hosmer-Lemeshow goodness-of-fit test for 10 groups was applied and the area under the Receiver Operating Characteristic (ROC) curve calculated to test the models’ fit [40,44].

## Results

The mean age of female respondents (41.5 years, SD 15.2) was slightly but significantly lower than that of male respondents (43.9 years, SD 16.6). Table 1 provides the distribution of selected characteristics among female and male population samples and the weighted estimates for Armenia. The prevalence of fair/poor self-rated health was equally high in both genders (61.8% among women and 59.7% among men). The risk of depression was more prevalent among women. Men reported incomplete school or university/higher education more often. The proportion of employed was higher among men. They also reported significantly more smoking and alcohol consumption. No other significant differences were found between the genders.

**Table 1:** **Distribution of selected social structure, behavioral/attitudinal and psychosocial variables among women and man **^**a**^

	**Women (%)**	**Men (%)**
Self-rated health	*n=2297*	*n=452*
Excellent, very good, good	38.2 (38.3)	40.3 (43.7)
Fair, poor	61.8 (61.7)	59.7 (56.3)
Physical health	*n=1839*	*n=401*
Severe health problems	33.4 (33.3)	29.2 (28.2)
Moderate health problems	30.9 (30.9)	31.7 (30.1)
No health problems	35.7 (35.8)	39.2 (41.7)
Depression^**^	*n=1689*	*n=336*
Yes	41.7 (41.1)	29.8 (24.1)
No	58.3 (58.9)	70.2 (75.9)
Weak social support	*n=2027*	*n=411*
Yes	24.9 (24.1)	26.5 (24.6)
No	75.1 (75.9)	73.5 (75.4)
Urban/rural residence	*n=2310*	*n=462*
Urban	48.5 (65.2)	48.1 (64.3)
Rural	51.5 (34.8)	51.9 (35.7)
Ethnicity	*n=2277*	*n=458*
Armenian	98.6 (98.8)	98.9 (99.1)
Other	1.4 (1.2)	1.1 (0.9)
Education^*^	*n=2287*	*n=441*
Incomplete school (<10 years)	8.9 (7.5)	12.2 (8.4)
School or upper secondary (10–13 years)	74.1 (68.6)	66.0 (63.0)
University or higher	17.0 (23.9)	21.8 (28.6)
Employment^**^	*n=2304*	*n=460*
Employed	15.1 (16.2)	54.6 (55.7)
Unemployed	84.9 (83.8)	45.4 (44.3)
Poverty status	*n=2310*	*n=462*
Severe poverty	25.8 (21.8)	26.2 (20.0)
Moderate poverty	25.6 (23.9)	24.2 (25.8)
No poverty	48.7 (54.3)	49.6 (54.2)
Low affordability of healthcare	*n=2309*	*n=462*
Yes	13.1 (11.4)	13.4 (11.0)
No	86.9 (88.6)	86.6 (89.0)
Current smoking^**^	*n=2207*	*n=428*
Yes	1.7 (3.6)	60.7 (64.1)
No	98.3 (96.4)	39.3 (35.9)
Ever smoking^**^	*n=2214*	*n=433*
Yes	3.7 (6.9)	83.8 (90.4)
No	96.3 (93.1)	16.2 (9.6)
Drinking alcohol^**^	*n=2204*	*n=420*
Once a week or more	4.4 (3.7)	40.2 (39.3)
Less than once a week	95.6 (96.3)	59.8 (60.7)
Binge drinking^**^	*n=2220*	*n=430*
Ever experienced	2.8 (2.9)	27.2 (29.8)
Never experienced	97.2 (97.1)	72.8 (70.2)
Attitude toward healthy lifestyle	*n=2085*	*n=414*
Positive	52.7 (56.7)	47.8 (47.8)
Indifferent/negative	47.3 (43.3)	52.2 (52.2)

^a^ Valid percentages among female and male population samples and weighted estimates for Armenia in parentheses.

^**^ Significant difference between female and male respondents at p<0.01 level. ^*^ Significant difference between female and male respondents at p<0.05 level. *n* – number of valid responses.

To affirm the use of self-rated health as a measure of health status, we first investigated its association with physical and mental health correlates – physical health and depression. For both genders, these correlates were strongly associated with the outcome. The associations remained highly significant when controlling for these variables mutually and concurrently for all other significant variables. The effect of such control was minimal for the association between the outcome and physical health (Table 2). In contrast, the strength of association between self-rated health and depression was relatively weaker and weakened further when controlling for other variables. This finding suggests that self-rated health more closely reflects physical health, although still capturing important aspects of mental health.

**Table 2:** **The association of fair/poor self-rated health with physical health and depression among women and men **^**a, b**^

**Variable/category**	** Model 1**		** Model 2**	
	**OR**	**95% CI**	**OR**	**95% CI**
*Women*				
Health problems				
Severe	22.19	15.20–32.38	18.40	11.66–29.03
Moderate	3.66	2.85- 4.62	3.58	2.68–4.80
Minimal/none (ref.)	1.00		1.00	
Depression	3.36	2.66–4.25	2.03	1.51–2.73
*Men*				
Health problems				
Severe	36.83	14.49–93.58	44.30	13.71–143.22
Moderate	3.62	2.15–6.08	5.48	2.80–10.71
Minimal/none (ref.)	1.00		1.00	
Depression	3.36	1.90–5.95	2.51	1.16–5.40

^a^ Odds ratios (OR) with 95% confidence intervals (CI).

^b^ Model 1: controlled for age. Model 2: controlled for significant variables in all three dimensions and for physical health.

In bivariate regression analysis, the associations between fair/poor self-rated health and urban/rural residence, ethnicity, current smoking, ever smoking and binge drinking were insignificant for both genders. Low affordability of healthcare and positive attitude toward healthy lifestyle were significantly related to self-rated health only among women and moderate drinking – only among men. For both samples, the insignificant variables were excluded from the multivariate analysis.

Table 3 presents the association between fair/poor self-rated health and the studied variables among women. After controlling only for age (Model 1), fair/poor self-rated health was significantly associated with respondent’s age, employment, education (secondary), poverty (severe and moderate with dose–response relation), low affordability of healthcare, depression and weak social support. Only employment demonstrated a protective effect, suggesting that employed women were less likely to report fair/poor health. When controlling for all social structure variables (Model 2), education and moderate poverty became insignificant, while the others remained significant. All these associations weakened but remained significant in Model 3, which controlled for significant variables in all three dimensions.

**Table 3:** **The association of fair/poor self-rated health with social structure, behavioral/attitudinal and psychosocial variables among women **^**a, b**^

**Variable/category**	** Model 1**		** Model 2**		**Model 3 (Final Model: n=2310, valid n=1606)**	
	**OR**	**95% CI**	**OR**	**95% CI**	**OR**	**95% CI**
Age (years)	1.06	1.05–1.07	1.06	1.05–1.06	1.05	1.04–1.06
Employment	0.60	0.47–0.76	0.70	0.54–0.91	0.72	0.53–0.99
Education						
Less than school (<10 years)	1.30	0.85–1.98	0.82	0.52–1.28	0.60	0.34–1.05
Secondary (10–13 years)	1.53	1.21–1.94	1.18	0.92–1.53	1.21	0.90–1.64
University or more (ref.)	1.00		1.00		1.00	
Poverty						
Severe	2.16	1.70–2.74	1.82	1.41–2.34	1.40	1.01–1.94
Moderate	1.30	1.05–1.62	1.17	0.93–1.46	1.16	0.88–1.53
No poverty (ref.)	1.00		1.00		1.00	
Low affordability of healthcare	2.49	1.82–3.41	2.14	1.55–2.95	1.95	1.28–2.98
Depression	3.36	2.66–4.25			2.75	2.14–3.53
Weak social support	2.30	1.80–2.94			1.90	1.42–2.56
Positive toward healthy lifestyle	0.90	0.74–1.08				
*Hosmer& Lemeshow goodness of fit test*					*p=0.390*	
*Area under ROC curve*					*0.780*	
*Pseudo R*^*2*^					*0.179*	

^a^ Odds ratios (OR) with 95% confidence intervals (CI).

^b^ Model 1: controlled for age. Model 2: controlled for significant variables in social structure (age, employment, education, poverty, low affordability of healthcare). Model 3: controlled for significant variables in all three dimensions. Final Model: fitted model.

Table 4 demonstrates the associations between fair/poor self-rated health and its possible determinants among men. When controlling only for age (Model 1), significant associations were detected for education (with dose–response relation), severe poverty, depression, weak social support and moderate drinking. Only the latter demonstrated protective effect – those who reported moderate drinking were less likely to rate their health as “fair/poor”. The associations remained significant after controlling for all social structure variables (Model 2). However, after also controlling for the variables in psychosocial and behavioral/attitudinal dimensions (Model 3), severe poverty lost its significance.

**Table 4:** **The association of fair/poor self-rated health with social structure, behavioral/attitudinal and psychosocial variables among men **^**a, b**^

**Variable/category**	**Model 1**		**Model 2**		**Model 3**		**Final Model: n=462, valid n=291**	
	**OR**	**95% CI**	**OR**	**95% CI**	**OR**	**95% CI**	**OR**	**95% CI**
Age (years)	1.03	1.02–1.05	1.03	1.02–1.05	1.04	1.02–1.06	1.04	1.02–1.06
Employment	0.75	0.49–1.13						
Education								
Less than school (<10 years)	2.74	1.26–5.95	2.33	1.05–5.16	2.75	1.04–7.30	2.95	1.14–7.60
Secondary (10–13 years)	1.96	1.19–3.23	1.75	1.04–2.95	2.34	1.19–4.60	2.50	1.32–4.74
University or more (ref.)	1.00		1.00		1.00		1.00	
Poverty								
Severe	2.22	1.32–3.73	1.95	1.14–3.33	1.07	0.52–2.20		
Moderate	1.38	0.84–2.26	1.18	0.70–1.98	1.28	0.66–2.47		
No poverty (ref.)	1.00		1.00		1.00			
Depression	3.36	1.90–5.95			3.18	1.69–5.99	3.18	1.71–5.91
Weak social support	2.26	1.33–3.82			2.89	1.45–5.73	2.88	1.48–5.60
Drinking once a week or more	0.65	0.42–0.99			0.58	0.33–1.01	0.57	0.33–0.99
*Hosmer & Lemeshow goodness of fit test*							*p=0.802*	
*Area under ROC curve*							*0.767*	
*Pseudo R*^*2*^							*0.174*	

^a^ Odds ratios (OR) with 95% confidence intervals (CI).

^b^ Model 1: controlled for age. Model 2: controlled for significant variables in social structure (age, education, poverty). Model 3: controlled for significant variables in all three dimensions. Final Model: fitted model.

The final fitted model for women was the same as Model 3 and revealed six independent predictors of fair/poor self-rated health: age, employment, severe poverty, low affordability of healthcare, depression and weak social support (although education was insignificant, we kept it in the final model to achieve better model fit) (Table 3). Depression was the strongest predictor – depressed women were almost three times more likely to report fair/poor health. The next strongest predictors in women were weak social support and low affordability of healthcare. These factors increased the likelihood of perceived fair/poor self-rated health almost two times. Severely poor women were 1.4 times more likely to report fair/poor self-rated health. Employment was the only variable with protective effect on women’s perceived health.

For men, the final fitted model identified five significant predictors of perceived fair/poor health, of which three were the same as for women: age, depression and weak social support (Table 4). Again, the strongest association was found for depression (OR 3.18), closely followed by weak social support (OR 2.88). The next strong predictor among men was education: those with less than secondary education had three times the odds and those with secondary education 2.5 times higher odds of reporting fair/poor health compared to the group with higher education. Moderate drinking was inversely associated with the outcome among men: those who reported moderate drinking had 43% reduced odds of rating their health as fair/poor.

The final models were checked for multicollinearity and the detected highest value for the Variance Inflating Factor did not exceed 1.41, indicating no issue of collinearity [45]. For both genders, the final models had acceptable calibration and discrimination (Tables 3, 4).

## Discussion

The prevalence of fair/poor self-rated health found in this study was rather high with no significant between-gender differences: 59.7% among men and 61.8% among women. These proportions exceed those reported in 1990s and 2000s in central and western European countries [46,47], but are comparable to those in Baltic countries (56–66% among men and 64–68% among women) [29], and are lower than in Russia (61.6% and 78.9%, respectively) [48]. Women tend to report fair/poor health more often than men [20,28,47-49]. However, between gender differences in self-rated health are frequently insignificant [2,21,50,51]. A recent study from Sweden suggests that gender differences in self-rated health would disappear if women were as secure financially as men and were not treated in a condescending manner to a larger extent than men [49].

The proportion of women with fair/poor self-rated health in this study is considerably lower than the previously reported numbers from Armavir *marz,* Armenia (80.0% in 2001) [35]. For better comparability with these numbers, we examined the prevalence of fair/poor self-rated health in the subsample of women from Armavir *marz* in this study and found even lower proportion (53.4%), which indicated a positive dynamic in this indicator in Armavir *marz* and possibly in Armenia from 2001 to 2006. According to the country’s official statistics, this period was characterized by economic growth and corresponding decline in the proportions of both extremely poor (from 16.0% in 2001 to 4.1% in 2006) and poor (from 34.9% to 26.5%) [33]. The Armavir study found that material deprivation was the strongest predictor of poor self-rated health, with a clear dose–response relationship [35]. In this study, the effect of poverty on self-rated health was mediated largely by psychosocial variables. Thus, in the final model, poverty remained an independent predictor of fair/poor self-rated health only among women. A growing body of evidence showed that the association between material conditions and health outcomes intensifies as income inequality increases and that a threshold of income inequality exists beyond which its negative impacts on health begin to emerge [38]. The importance of psychosocial pathways through which material circumstances affect health indirectly also is well recognized [27] and can partly explain our findings.

In the final fitted models for both genders, the strongest association with fair/poor self-rated health was found for psychosocial variables – being at risk for depression and reporting weak social support. Previous studies well documented the detrimental effect of depression on health [7,30,52,53]. Similar to this study, a study from Hungary found depression to be a stronger predictor of self-rated health than socio-economic deprivation and mediate between the latter and self-rated morbidity rates, especially among men [30]. The study authors hypothesized that in suddenly changing societies material deprivation and depressive symptomatology could worsen each other creating a vicious cycle that leads to higher self-rated morbidity rates.

Social support was found to be protective of health in many studies [7,22,25,53-58]. Different emotional and instrumental aid pathways through which social support might influence health were suggested, including better coping abilities with stress, higher sense of self-esteem, self-efficacy and coherence, positive feelings of belonging and attachment, more engagement in health-promoting behaviors and refraining from health-damaging ones [59]. We found greater impact of weak social support on perceived health for men than for women, a finding consistent with that from the French Gazel cohort study [53].

For men, education also showed strong independent dose–response association with self-rated health. This is a common finding in many transition countries [19,29]. However, this association in transition countries is weaker than that seen in the west [60]. The most common explanation for this difference is that the link between education, occupation and material well-being in these countries is not as consistent as in the west [2,20,61]. Thus, in countries like Armenia, better knowledge of health and better copping abilities among educated might play a more important role in the observed positive association between education and health than a secure position in the labor market that higher education usually guarantees in the west [2].

We found that educational level was independently associated with self-rated health among men, but not among women. This finding is consistent with other studies, suggesting that educational level has stronger health effect in men than in women [22,61]. Instead, material conditions, affordability of healthcare and employment were found to be independent predictors of self-rated health among women, but not men. These factors are known determinants of self-rated health [21,23,24,32,38]. However, their independent effect on women’s perceived health can be explained by the fact, that women are more engaged in household duties than men [61,62]. Together with unemployment that affected women disproportionately more in Armenia [33], this reality places women in a situation where the influence of household-related factors on their health is perhaps stronger than that of outside factors. A number of studies have shown the independent effect of the amount of household labor and housing attributes on women’s perceived health [43,62,63]. The influence of housing factors supersede the effect of educational attainment on perceived health [63]. In this study, we did not measure the effect of housing and household labor on women’s self-rated health directly. However, poverty and low affordability of healthcare can serve as indirect measures of poor housing conditions making women’s household labor more strenuous for many reasons including lack of resources and amenities to facilitate the labor.

Previous research has repeatedly shown the relation of unemployment with poor health [22,24,26,32]. Unemployment can influence health through different pathways including reduced income, psychosocial stress and loss of social networks. In transition periods, however, when unemployment is a widespread phenomenon, the psychosocial stress caused by it becomes less pronounced [64]. This latter explanation is consistent with the situation in Armenian and our finding for men that unemployment was not an independent predictor of fair/poor perceived health. Employment might contribute differently to perceived health status for women in Armenia. Employment allows women partially to trade household duties for greater control over their life, which was shown to be associated with better perceived health [2,19,20,28,56]. Unemployed women, however, are fully dependent on their housing environment.

This study revealed a protective effect of moderate drinking on self-rated health among men. Previous studies have found a U-shaped relation between alcohol consumption and health: heavy drinkers and those with past history of alcohol abuse usually reported poor health, while those consuming small to moderate amounts of alcohol were more likely to report better health than abstainers [3,14,48,65]. The positive effect of moderate alcohol consumption on health was attributed to its ability to reduce the risk of cardiovascular and cerebrovascular adverse health events [65]. However, in cross-sectional studies, a reverse causation is also a possibility, when poor health prevents drinking. In a study in Russia, frequent drinking was linked to better self-rated health but was also predictive for higher mortality [48].

Our study did not find any other independent relations between self-rated health and behavioral/attitudinal variables. These results are consistent with previous studies, which also have shown that behavioral factors usually explain only a small portion of the socioeconomic differences in self-rated health [66,67].

### Study limitations

The refusal rate in this household health survey (21.1%) was in the acceptable range for population-based studies. The characteristics of those who refused to participate are unknown, but it is unlikely that response bias could affect the results. Although the sampled households were representative for each *marz*, the respondents were selected to preferably include married women or those having children less than 18 years and their husbands. This method could have resulted in an under-representation of older age groups. We compared the age structure of our sample with that of Armenia’s general population [33] and found slight over-representation of middle aged (25–40 years old) and slight under-representation of older aged (65 and over) in our female sample. In the male sample, younger men (under 29) were slightly underrepresented, while older men (65 and over) were slightly overrepresented. This modest disparity might have hindered our ability to find between-gender differences in the prevalence of fair/poor self-rated health. However, age-adjusted logistic regression analysis of the whole sample (not shown) did not indicate significant between-gender differences in self-rated health.

The cross-sectional design of this study makes drawing conclusions on which factors are determinants and which are consequences of fair/poor self-rated health impossible. However, the set of factors independently associated with self-rated health in this study largely mirrors those identified by a number of longitudinal studies as determinants of ill health [7,14,24,38,53,54,65].

As the study was based on the secondary analysis of previously collected data, our choice of independent variables was limited to what these data could provide. Hence, several important potential predictors of self-rated health like perceived life control, details of employment and household labor were left out from this study.

## Conclusions

Although the prevalence of fair/poor self-rated health was not different among men and women in this study, the sets of independent predictors of perceived health were somewhat different. In western societies, causes of health inequalities are generally similar for both genders [51]. In Armenia, however, the lifestyles of women and men are more different than in the west. Household-related duties are more dominant for women, while men are more engaged in activities outside the household. Most probably, the identified between-gender differences in the sets of predictors of perceived health reflect these realities. Nevertheless, this study found no such differences in terms of the strongest predictors of perceived health – psychosocial variables. For both genders, depression and lack of social support were the strongest predictors of fair/poor self-rated health.

This study showed that since the Armavir study in 2001 [44], the set of factors independently associated with self-rated health have changed. The role of material deprivation decreased while the influence of psychosocial factors on perceived health became dominant. The economic growth in Armenia during 2001–2006 could partially explain this change, as it resulted in some reduction of poverty. Another explanation is that in the situation, when poverty became less severe, material circumstances affected health indirectly through psychosocial pathways. The findings of this study emphasize the importance of social reforms to further reduce poverty, ensure universal access to basic healthcare services and improve the psychosocial environment in Armenia. Given the evidence that former socialist countries of Central and Eastern Europe and the former Soviet Union share common features in terms of the positive influence of open/uncorrupted infrastructures and income per capita on their population’s health [31,68,69], the recommendations of this study could be relevant to other economies in transition.

## Competing interests

The authors declare that they have no competing interests.

## Authors' contributions

All authors participated in conceptualizing the original assessment and designing the survey instrument. A. Demirchyan conceptualized the specific research questions, performed the analysis and drafted the manuscript. V. Petrosyan and M.E. Thompson substantially contributed to the interpretation of data and critically revised the manuscript. All authors read and approved the final manuscript.
